# Old Soil Organic Matter Has a Low Carbon:Nitrogen Ratio—A Global Meta‐Analysis About ^14^C and Soil Stoichiometry

**DOI:** 10.1111/gcb.71107

**Published:** 2026-09-21

**Authors:** Marie Spohn

**Affiliations:** ^1^ Department of Soil and Environment Swedish University of Agricultural Sciences (SLU) Uppsala Sweden

**Keywords:** ^14^C, carbon:nitrogen ratio, element cycling, land cover, persistence, radiocarbon, soil C:N ratio, soil depth, soil organic matter, soil stoichiometry

## Abstract

Soil organic matter (SOM) persistence and the SOM carbon‐to‐nitrogen (C:N) ratio determine long‐term nitrogen (N) sequestration in terrestrial ecosystems. Yet, the question of how SOM persistence is related to the C:N ratio of SOM is still not fully resolved. Based on a global dataset on Δ^14^C in soils, I tested the hypothesis that SOM persistence is negatively correlated with the C:N ratio because N is enriched relative to carbon (C) as SOM ages. The results of this study demonstrate that the soil Δ^14^C decreased with decreasing C:N ratio, indicating that old, persistent SOM has a low C:N ratio, as hypothesized. The relationship between Δ^14^C and the C:N ratio had a breakpoint at a C:N ratio of approximately 13, indicating that organic matter with a high C:N ratio decomposes quickly, while SOM with a C:N ratio below 13 decomposes substantially more slowly. The Δ^14^C was higher in forest and shrubland soils than in grassland and cropland soils throughout the soil profile. High soil Δ^14^C values were associated with high soil C:N ratios in topsoil layers of forests and shrublands, suggesting that high inputs of plant detritus with high C:N ratios keep the C:N ratio elevated in the topsoil layers of these ecosystems. In conclusion, this study shows that old SOM has a low C:N ratio, indicating that organic N is preferentially retained in soils compared to organic C. The results have important implications for element cycling and soil C sequestration because they show that N is stored on average for longer periods in soils compared to C and that an increase in the amount of organic C that is sequestered in soils over centuries would strongly increase the amount of sequestered N.

## Graphical abstract

This study demonstrates that old SOM has a low C:N ratio compared to young SOM, indicating that N is preferentially retained in soils compared to organic C. Furthermore, the study shows that the C:N ratio and the Δ^14^C are higher in forest and shrubland soils than in grassland and cropland soils throughout the soil profile.

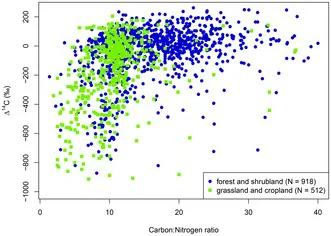

## 1 Introduction

Soil organic matter (SOM) forms a major pool of carbon (C) and nitrogen (N) in the Earth system that is important for soil fertility and Earth's climate (Batjes 2014; Friedlingstein et al. 2025). Together, SOM persistence and the C:N ratio of SOM determine long‐term N sequestration in terrestrial ecosystems as well as long‐term N supply for primary production. In addition, the N content of SOM affects C storage in soils. Yet, the question of how the persistence of SOM is related to the C:N ratio is still not fully resolved (Knicker 2011; Spohn 2024).

Soil organic N is mostly composed of proteins, peptides, amino acids, amino sugars, and heterocyclic compounds (Knicker 2014). Thus, soil organic N has a high density of functional groups, such as amino and carboxyl groups, that have a high affinity to adsorb to mineral surfaces. In addition, proteins have a high affinity to interact with mineral surfaces, for example through hydrophobic interactions (Haynes and Norde 1994; Kleber et al. 2007; Knicker 2011; Yu et al. 2013; Spohn 2024). Consequently, mineral‐associated organic matter is typically very rich in organic N (Kleber et al. 2007; Cotrufo et al. 2019; Amorim et al. 2022). The adsorption of organic N to mineral surfaces likely leads to an enrichment of organic N relative to organic C during the decomposition of organic matter because organic N compounds are preferentially stabilized due to their interactions with mineral surfaces (Kleber et al. 2007; Knicker 2011; Yu et al. 2013; Spohn 2024). Thus, it can be hypothesized that the C:N ratio decreases with SOM age since N‐containing organic compounds have a high affinity to adsorb to mineral surfaces, which likely stabilizes them against decomposition and enriches N relative to C in SOM over time.

The relationship between SOM persistence and SOM stoichiometry can be studied using radiocarbon measurements combined with soil element analyses. Radiocarbon measurements determine the ratio of ^14^C:^12^C (Δ^14^C), which decreases with increasing age of organic matter due to radioactive decay. Thus, the longer C has persisted in an ecosystem since the time of fixation from the atmosphere, the lower the Δ^14^C (Trumbore 2009).

The Δ^14^C and the C:N ratio of SOM both depend on organic matter inputs to soil. Organic matter inputs, i.e., plant detritus and root exudates, have been enriched with ^14^C during the last decades. The reason for this enrichment is thermonuclear weapons tests conducted in the 1950s and early 1960s which strongly increased the amount of ^14^C–CO_2_ in the atmosphere (Trumbore 2009). Thus, it can be expected that soils with large annual inputs of plant detritus have a higher Δ^14^C than soils with small organic matter inputs, during the decades following the thermonuclear weapon tests. Organic matter inputs to soils are typically higher in forests than in grasslands due to the high primary production in forests (Lieth 1973; Knapp and Smith 2001), and they are higher in grasslands than in croplands due to the removal of aboveground biomass during harvest of croplands (Janzen 2015). Thus, cropland soils can be expected to have a lower ^14^C incorporation than forest soils, during the decades following the thermonuclear weapons tests. Plant detritus inputs, and specifically their C:N ratios also affect the C:N ratio of SOM. The C:N ratio of both plant detritus and SOM usually decreases in the order forest > shrubland > grassland > croplands. Yet, the C:N ratio of soils is on average substantially lower than the C:N ratio of plant detritus (Xu et al. 2013; Zechmeister‐Boltenstern et al. 2015).

How the soil Δ^14^C is related to the soil C:N ratio as the result of organic matter inputs and decomposition is still not fully understood. During the last decade, the number of Δ^14^C measurements of SOM has increased due to the advent of the Mini Carbon Dating System (MICADAS), which made ^14^C measurements more affordable. This has created an opportunity to better understand the relationship between SOM stoichiometry and SOM persistence.

Here, I hypothesize that the soil Δ^14^C is positively correlated with the soil C:N ratio. The reason for this is twofold: first, SOM with a low C:N ratio can be expected to be older; second, in ecosystems in which SOM has a high C:N ratio, such as forests, organic matter inputs to the soil tend to be high (as explained above). To test this hypothesis, I conducted a global meta‐analysis based on the International Soil Radiocarbon Database.

## 2 Material and Methods

### 2.1 Dataset

This study is based on data from the International Soil Radiocarbon Database (ISRaD, version 2.9.9, released in August 2025; Lawrence et al. 2020; Beem‐Miller et al. 2021; von Fromm et al. 2026). Specifically, I analyzed data on the bulk soil of soil horizons and depth increments (called soil layers in the following). The database reports the soil organic Δ^14^C, which is corrected for the radioactive decay of the oxalic acid standard between 1950 and the measurement year. To account for mass‐dependent fractionation effects, the reported ^14^C:^12^C ratio of all samples is corrected to a common δ^13^C value of −25‰ (Stuiver and Polach 1977). Positive Δ^14^C values indicate incorporation of bomb‐radiocarbon produced by nuclear‐bomb tests in the 1950s and 1960s. Negative values indicate that most C entered the soil before the 1950s and enough time has passed for substantial radioactive decay to occur. The longer C has persisted in soil since the time of fixation from the atmosphere, the lower the Δ^14^C. The database reports the C:N ratio calculated based on mass.

For this study, I selected all soil layers of the database that contain data on both Δ^14^C and the soil C:N ratio. Observations of soils of the groups Andisol, Histosol, and Gelisol were excluded, similar as in previous studies (e.g., von Fromm et al. 2024). I excluded Andisols (i.e., volcanic soils) because they often contain charcoal that has been mixed with the soil parent material before the beginning of pedogenesis. Furthermore, I excluded Histosols (i.e., peatland soils) and Gelisols (i.e., permafrost soils) because in these soils, SOM is mostly preserved due to water logging (Histosols) or freezing (Gelisols), in contrast to other soil groups. Furthermore, I excluded 17 observations from the Arctic (Svalbart) since these soils are also frozen during most of the year, and one study that contributed nine observations, of which five were strong outliers. From this dataset, I excluded very few (strong) outliers by excluding observations with a C:N ratio < 1 or > 40.

After this selection, the final dataset contained data on 1430 soil layers, derived from 57 studies. Of the 1430 soil layers, 819 were from forests, 337 from grasslands, 175 from croplands, and 99 from shrublands. Furthermore, of the 1430 soil layers, 120 had a total organic carbon (TOC) content above 150 g kg^−1^ and were organic horizons of forest soils. Half of the data was collected between 2017 and 2024, and 75% between 2009 and 2024 (Figure S1). Thus, I did not correct for potential temporal changes as the decline in atmospheric Δ^14^C during the study period has very likely only a small effect on the results (for a meta‐analysis on soil radiocarbon that chose the same approach see von Fromm et al. (2024)). For 556 of the 1430 soil layers, data on pH (measured in water) was available.

### 2.2 Data Analyses and Statistics

The mean soil depth was calculated as the sum of the top and the bottom depth divided by two, for each soil layer. I conducted regression analysis and piecewise regression analysis using the R package segmented (version 2.2.9), whereby regressions with a *p* value < 0.05 were considered statistically significant. Furthermore, I conducted the student's *t*‐test whereby differences with a *p* value < 0.05 were considered statistically significant. All analyses were conducted in R (version 4.4.1, R Core Team 2021). Quantitative comparisons of radiocarbon contents were made based on the F notation and not based on the delta (Δ) notation.

## 3 Results

**FIGURE 1:**
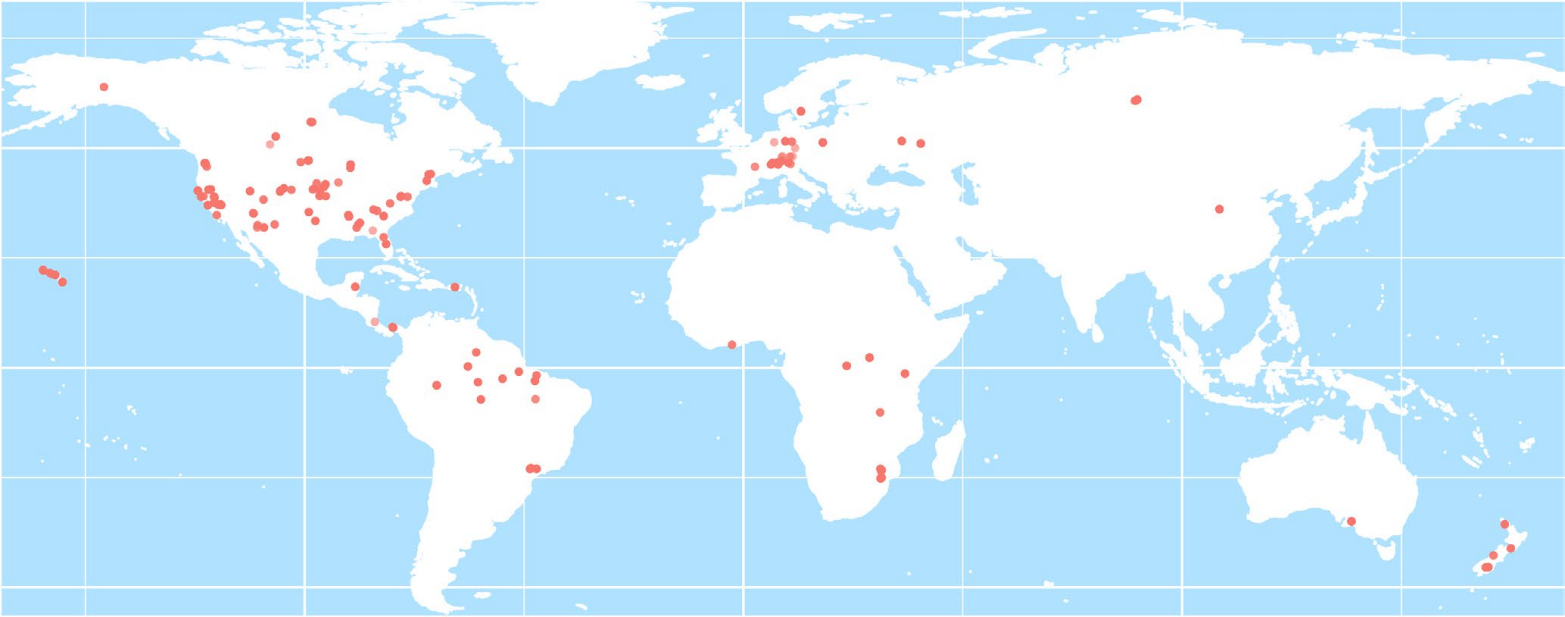
Map showing the locations of the study sites (red dots).

The dataset comprises 1430 observations from Africa, Asia, Europe, North America, Oceania, and South America (Figure 1). The median C:N ratio was 12.1, and the first and third quartiles of the C:N ratio were 10.0 and 14.3, respectively. The median Δ^14^C was −26 per mil, and the first and third quartiles of Δ^14^C were −169 and 50 per mil, respectively.

**FIGURE 2:**
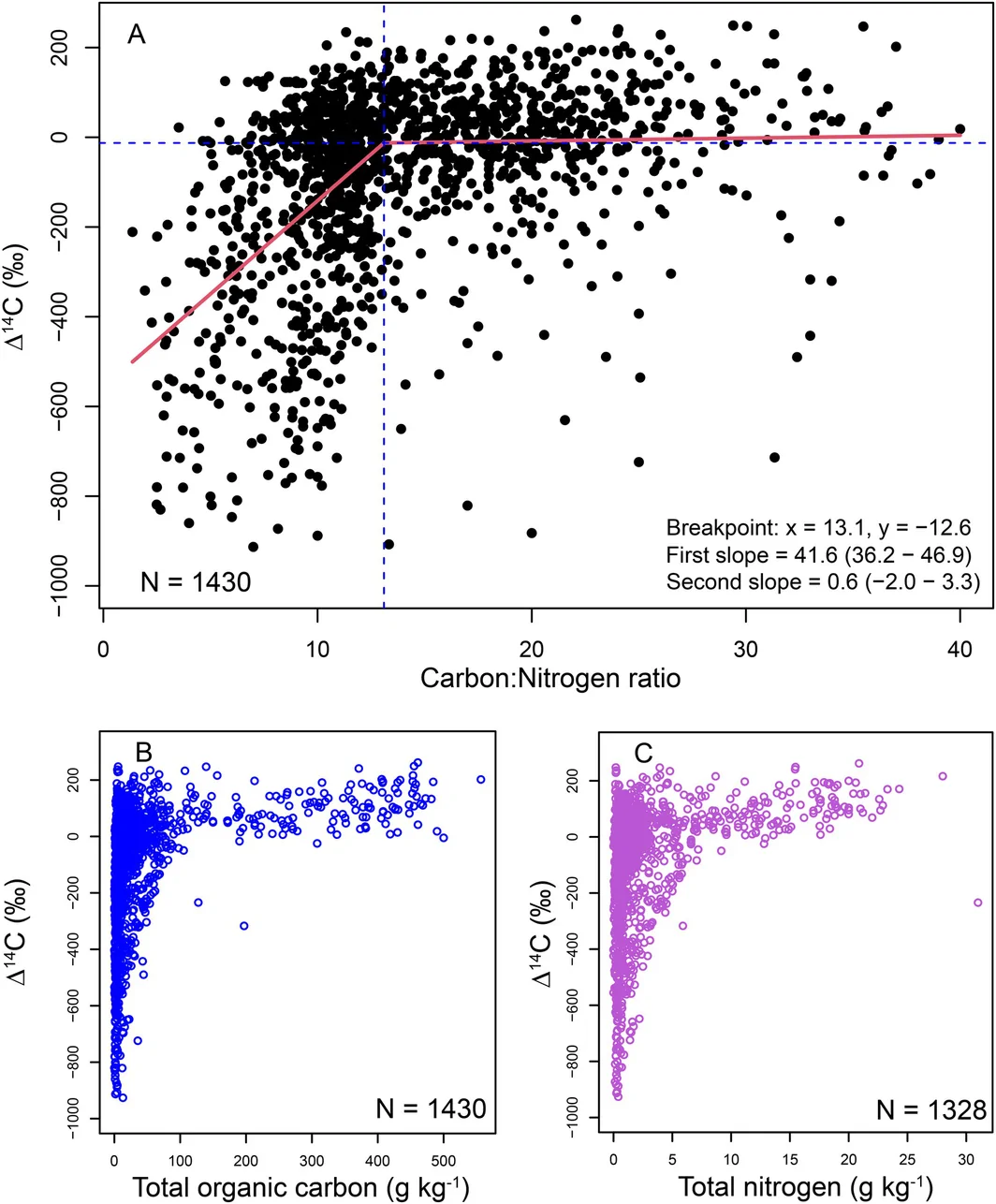
The Δ^14^C as a function of the C:N ratio (A) and as a function of the total organic carbon (B) and total nitrogen (C) contents. N indicates the number of observations.

The Δ^14^C was positively, linearly correlated with the C:N ratio (*p* < 0.001, *R* ^2^ = 0.14). However, the relationship between Δ^14^C and the C:N ratio had a breakpoint at a C:N ratio of 13.1 and a Δ^14^C of −12.6 per mil, as indicated by piecewise regression analysis (Figure 2A; *p* < 0.001, *R* ^2^ = 0.24). Below the C:N ratio of 13.1, the slope of the regression was 41.6 (lower 95% confidence interval (CI) = 36.2 and upper 95% CI = 46.9). Above 13.1, the slope of the regression was 0.6 (lower 95% CI = −2.0 and upper 95% CI = 3.3; Figure 2A). The Δ^14^C also tended to be lower in soil layers with lower TOC and total N concentrations (Figure 2B,C). However, there was not such a clear breakpoint as for the C:N ratio (Figure 2), not even when the 120 organic layers of the forest soils were excluded.

**FIGURE 3:**
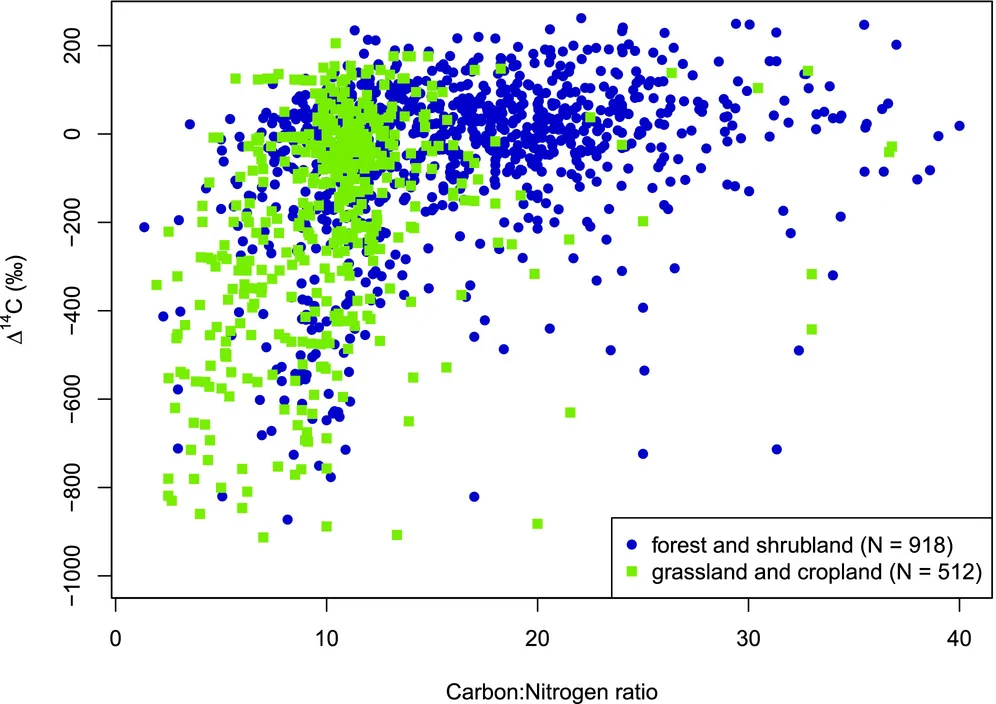
The Δ^14^C as a function of the soil C:N ratio of ecosystems with woody vegetation (forests and shrublands) and non‐woody vegetation (grasslands and croplands).

**FIGURE 4:**
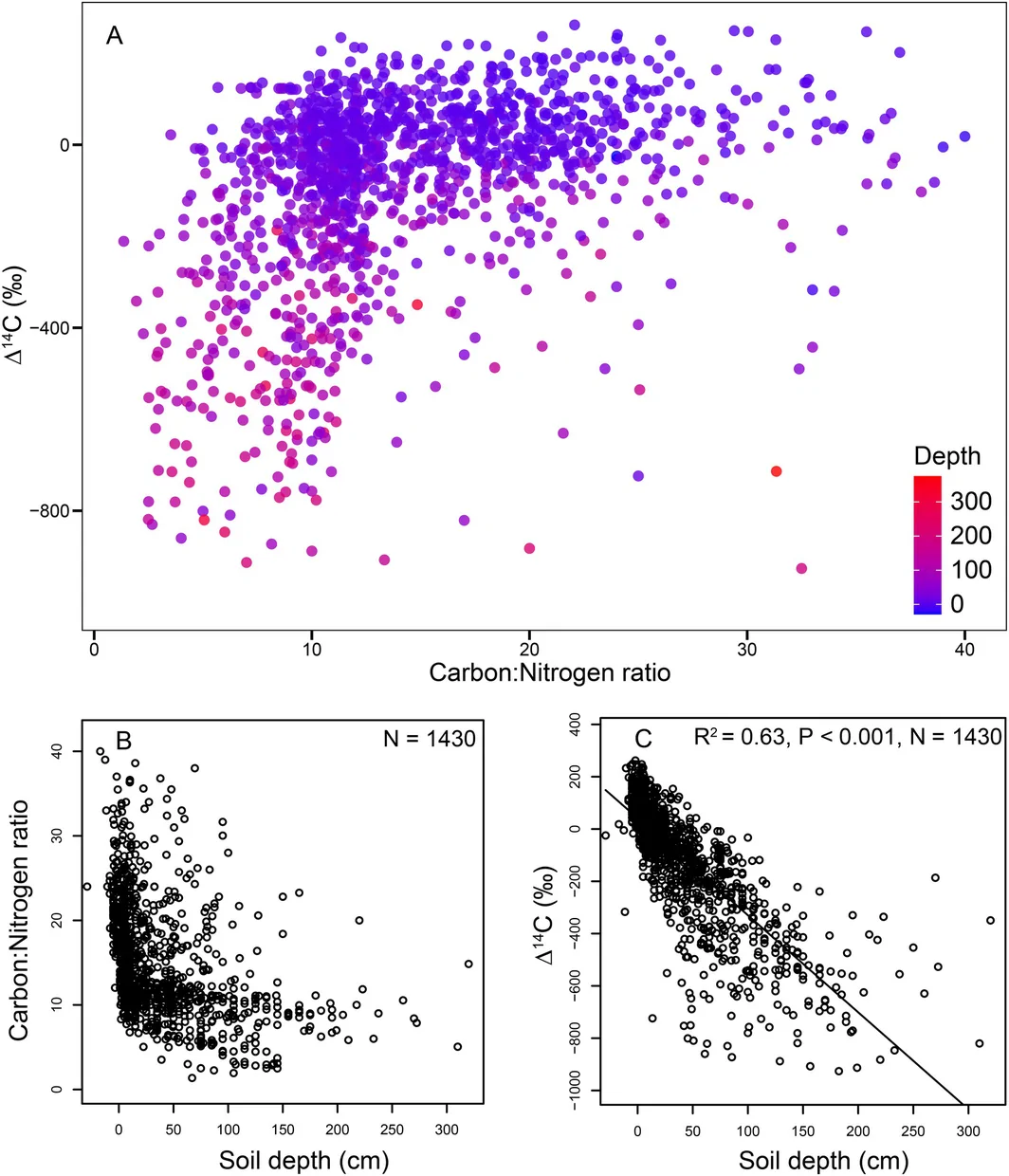
Δ^14^C as a function of the C:N ratio whereby soil depth is indicated by color (A), together with the C:N ratio (B) and Δ^14^C (C) as a function of soil depth. N indicates the number of observations.

Low C:N ratios were associated with low Δ^14^C in all ecosystems (Figure 3 and Figure S2). Many forest and shrubland soil layers had C:N ratios above 17 and positive Δ^14^C values, in contrast to the grassland and cropland soils (Figure 3 and Figure S2). SOM with very low Δ^14^C (i.e., < −250 per mil) and low C:N ratio (i.e., < 12) was mostly found in the deep soil below 100 cm (Figure 4A). Soil Δ^14^C decreased linearly with soil depth (Figure 4C, *p* = 0.001, *R* ^2^ = 0.63). In contrast, the C:N ratio decreased with soil depth in the uppermost cm but changed little with soil depth below 100 cm (Figure 4B). Furthermore, soil layers with very low Δ^14^C (i.e., < −250 per mil) in combination with low C:N ratio (i.e., < 12) tended to have higher pH values than the soil layers with higher C:N ratio (Figure S3).

**FIGURE 5:**
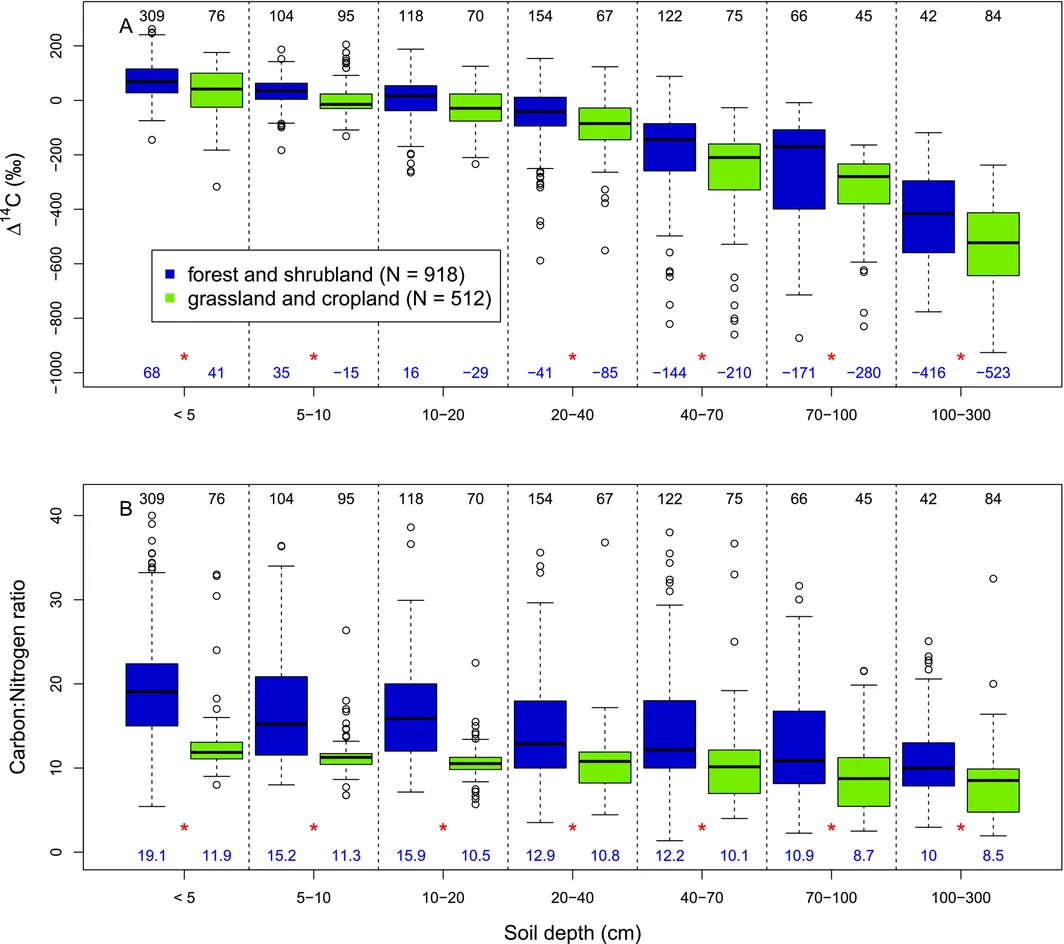
The Δ^14^C (A) and the C:N ratio (B) in different soil depth increments in ecosystems with woody vegetation (forests and shrublands) and non‐woody vegetation (grasslands and croplands). The black numbers indicate the numbers of observation. The blue numbers depict medians. The 25% and 75% percentile define the box and the 10% and 90% percentiles define the bars in the boxplots. Red asterisks indicate significant differences between ecosystems with woody and non‐woody vegetation.

Grassland and cropland soils combined (i.e., ecosystems with non‐woody vegetation) had on average a significantly lower Δ^14^C and a lower C:N ratio than forest and shrubland soils combined (i.e., ecosystems with woody vegetation), throughout the soil profile (Figure 5). The C:N ratio decreased more strongly with increasing depth in forest and shrubland soils than in grassland and cropland soils (Figure 5B). Specifically, the C:N ratio was on average higher in the topsoil compared to the lowest soil depth increment, by a factor of 1.9 in forests and shrublands, and by a factor of 1.4 in grasslands and croplands (Figure 5B). In addition, the radiocarbon signature of the soil layers was on average higher in the topsoil compared to the lowest soil depth increment, by a factor of 1.8 in forests and shrublands, and by a factor of 2.2 in grasslands and croplands (Figure 5A). Furthermore, the Δ^14^C tended to be higher in soil layers of grasslands than in soil layers of croplands, but this difference was only significant in the topsoil and not in the subsoil, which is related to the relatively low number of observations for the subsoil of croplands (Figure S4). When different soil depth increments were considered separately, the C:N ratio and the Δ^14^C were not significantly correlated (*p* > 0.05), except for the soil depth increment 70–100 cm.

## 4 Discussion

The results demonstrate that the soil Δ^14^C decreases with decreasing C:N ratio, showing that old, persistent SOM has a low C:N ratio. The relationship between Δ^14^C and the C:N ratio had a breakpoint at a C:N ratio of 13.1 and a Δ^14^C of −12.6 per mil. Furthermore, the Δ^14^C and the C:N ratio were higher in forest and shrubland soils than in grassland and cropland soils throughout the soil profile.

### 4.1 The C:N Ratio Decreases With Increasing SOM Age

In accordance with the hypothesis, the results demonstrate that old SOM has a low C:N ratio (Figure 2A). The reason for this is most likely that organic N is rich in functional groups that allow it to adsorb to mineral surfaces (Haynes and Norde 1994; Yu et al. 2013; Spohn 2024). The adsorption of organic N to mineral surfaces stabilizes organic N against decomposition, as indicated by studies reporting that amino acids decompose more slowly than other organic compounds in soil (Miltner et al. 2009) and that interactions with minerals restrict protein decomposition and amino acid uptake by microorganisms (Chevallier et al. 2003; Vieublé Gonod et al. 2006; Hunter et al. 2016). Proteins persist for longer periods in soils than other compounds due to their capacity to bind rigidly to minerals (Demarchi et al. 2016). Thus, protein breakdown often represents a major bottleneck in the N cycle (Jan et al. 2009). Sorption of SOM to mineral surfaces is generally one of the most important mechanisms that stabilize SOM against decomposition (Kleber et al. 2015; Wiesmeier et al. 2019) because adsorbed compounds cannot be taken up by microorganisms. Sorption of organic N to mineral surfaces seems to lead to a preferential stabilization of N‐containing organic compounds in soils, as hypothesized earlier (Spohn 2024).

Besides adsorption of organic N to mineral surfaces, organic N might also persist in soils through microorganisms that retain organic N in their biomass. This might occur particularly in nutrient‐poor soils, where microorganisms recycle organic N compounds efficiently in their biomass, which increases the turnover time of N in the soil microbial biomass pool (Spohn 2016). Microorganisms can recycle N by re‐using amino acids and cell wall components instead of releasing these compounds (Johnson et al. 2013; Mayer et al. 2019). Yet, the retention of organic N in the biomass of microorganisms is likely less important than the interaction of organic N with minerals for the persistence of organic N in soils because the soil microbial biomass N stock is small (Xu et al. 2013) compared to the total stock of N in soils. Thus, only a small proportion of the total N can be retained in the microbial biomass.

SOM with very low Δ^14^C (i.e., < −250 per mil) and low C:N ratio (i.e., < 12) was mostly found in the deep soil below 100 cm (Figure 4A), indicating that SOM moves downward in the soil profile, while it ages and reduces its C:N ratio. Yet below 100 cm, the C:N ratio did not change further with increasing soil depth, suggesting that when SOM reaches a depth of approximately 100 cm, the SOM modifications that cause the decline in the C:N ratio slow down. The specific soil depth at which the C:N ratio stops decreasing in different soils depends very likely on climate and soil texture.

The relationship between Δ^14^C and the C:N ratio had a breakpoint (Figure 2A). The breakpoint is mainly caused by the combination of soil layers that have a very low C:N ratio and a low Δ^14^C signature (mostly located in the subsoil) and soil layers that have a very high C:N ratio and a rather high Δ^14^C signature (mostly located in the topsoil). The two different slopes of the relationship between the C:N ratio and the Δ^14^C indicate that the stoichiometry of SOM with a C:N ratio above 13 changes quickly during decomposition, whereas the stoichiometry of SOM with a C:N ratio below 13 changes only slowly. The reason for the quick initial decomposition is likely that organic matter with a high C:N ratio is rich in carbohydrates, cellulose, and hemicellulose that provide easily available energy to microorganisms. Carbohydrates, cellulose, and hemicellulose have low activation energies, allowing microorganisms to gain energy during the oxidation of these compounds (Williams and Plante 2018). Once the organic matter that has a low activation energy is oxidized, the C:N ratio decreases much more slowly, likely because it is energetically less rewarding for microorganisms to decompose organic matter with low C:N ratio consisting of compounds with higher activation energy and compounds that are adsorbed to minerals or coprecipitated with (oxy)hydroxides. These results are in line with the observation that the respiration rate per unit microbial biomass (called the metabolic quotient) decreases with decreasing C:N ratio (Spohn and Chodak 2015; Spohn 2015). During organic matter decomposition, the C:N ratio of SOM approaches the C:N ratio of microbial biomass, which is approximately 6 (Xu et al. 2013). The decline in the decomposition rate with decreasing C:N ratio suggests that the later stages of SOM decomposition are mostly driven by the microbial requirement for C rather than N since the C:N ratio is low compared to the microbial biomass C:N ratio, and thus C rather than N is likely to limit microbial metabolism.

### 4.2 The Δ^14^C Is Comparatively High in Forest and Shrubland Soils

The Δ^14^C was higher in forest and shrubland soils than in grassland and cropland soils throughout the soil profile (Figure 5), suggesting higher inputs of recently formed organic matter in forest and shrubland soils. The reason for this is likely the higher primary production of forests than of grasslands (Lieth 1973; Knapp and Smith 2001) and the removal of plant aboveground biomass in croplands that leads to low plant detritus inputs to cropland soils (Janzen 2015). The inputs of recently formed organic matter to soil include rhizodeposition as well as above‐ and belowground plant litter. In the subsoil, they also include recently formed organic matter inputs received from topsoil horizons via transport of solutes and bioturbation (Sierra et al. 2024). High soil Δ^14^C values associated with high soil C:N ratios in forest and shrubland soils suggest that high inputs of plant detritus with high C:N ratios keep the C:N ratio elevated in the topsoils of these ecosystems. In other words, the C:N ratio of the topsoil layers of forests and shrublands does not reach values as low as those of grassland and cropland soils due to the high input of plant detritus in combination with the high C:N ratio of these inputs. Furthermore, the comparatively high Δ^14^C values in the soils of the ecosystems with woody vegetation might also be because C stays on average relatively long in the vegetation of these ecosystems before entering the soil. Thus, a part of the C that was fixed in these ecosystems in the 1960s and 1970s, when the atmosphere was strongly enriched with ^14^C, entered the soils much later. In addition, the high Δ^14^C and the high C:N ratio in the soils of the ecosystems with woody vegetation are also related to slow decomposition of the organic matter in these ecosystems caused, among other factors, by the organic matter quality and the low pH (Figure S3).

### 4.3 Implications for Element Cycling and C Sequestration

The results of this study have important implications for element cycling and soil C sequestration. The slow decomposition of organic N relative to organic C, which causes the low C:N ratio in the subsoil, decreases the probability that N is efficiently recycled within the ecosystem since it increases the probability that N is lost from the ecosystem due to soil erosion and colloidal transport of N (Judy et al. 2018). The longer the period that N remains in the soil, the higher the risk that it is lost from the ecosystem due to erosion, which could cause N limitation of plants in the ecosystem. The sequestration of N over long periods in soil suggests that there are limits to efficient N recycling in ecosystems. Hence, the results of this study underline the importance of N inputs, such as N_2_ fixation, for maintaining N availability in terrestrial ecosystems.

The findings of this study suggest that sequestration of C in soils over long periods requires substantial amounts of N and that an increase in the amounts of C stored in soils over decadal to centennial timescales would strongly increase the amount of sequestered N. Hence, increased soil C sequestration might negatively impact the amount of N available to plants. This is important since N limits plant growth and especially crop growth in most parts of the world (LeBauer and Treseder 2008). The fact that soil C sequestration also leads to soil N sequestration has been discussed previously (e.g., van Groenigen et al. 2017). However, the results presented here highlight that specifically long‐term soil organic C sequestration, which could be relevant to combat climate change, is associated with a larger N retention than short‐term soil organic C sequestration.

## 5 Conclusions

This study demonstrates that old SOM has a low C:N ratio and that Δ^14^C and the C:N ratio are positively correlated. The observation that the C:N ratio increases with increasing age of SOM indicates that N is preferentially retained in soils compared to organic C. The two different slopes of the relationship between the C:N ratio and the Δ^14^C suggest that the stoichiometry of SOM with a C:N ratio above 13 changes quickly, while the stoichiometry of SOM with a C:N ratio below 13 changes only slowly. The long average retention time of N compared to C in soil has important implications for element cycling as it decreases the probability that N is efficiently recycled in the ecosystem. Furthermore, the results suggest that increasing the amount of organic C that is sequestered in soils over decadal to centennial timescales would strongly increase soil N sequestration.

## Author Contributions

**Marie Spohn:** conceptualization, investigation, writing – original draft, methodology, visualization, writing – review and editing.

## Funding

This work was supported by the European Research Council, 101043387.

## Conflicts of Interest

The author declares no conflicts of interest.

## Supporting information


**Figure S1:** Frequency of the calendar years of radiocarbon analysis (*N* = 1430).
**Figure S2:** Δ^14^C as a function of the C:N ratio. Colors indicate land cover.
**Figure S3:** Δ^14^C as a function of the C:N ratio. Colors indicate soil pH (determined in water). N indicates the number of observations.
**Figure S4:** The Δ^14^C in different soil depth increments in grasslands and croplands. The black numbers indicate the numbers of observation. The blue numbers depict medians. The 25% and 75% percentile define the box and the 10% and 90% percentiles define the bars in the boxplots. The red asterisk indicates a significant difference between grasslands and croplands.

## Data Availability

All data of this study are available in the International Soil Radiocarbon Database (ISRaD, version 2.9.9). https://github.com/International‐soil‐radiocarbon‐database/israd/.
