# Supplementary material for: Old Soil Organic Matter Has a Low Carbon:Nitrogen Ratio—A Global Meta‐Analysis About 14C and Soil Stoichiometry
Source: Glob Chang Biol. 2026 Sep 21;32(9):e71107. doi: 10.1111/gcb.71107 (PMC13594686; doi:10.1111/gcb.71107)
Supplement: Supplementary file 1 [file GCB-32-e71107-s001.pdf]

Supplement

Old soil organic matter has a low carbon:nitrogen ratio - A global meta-analysis about ^{14}C and soil stoichiometry

Marie Spohn

Department of Soil and Environment, Swedish University of Agricultural Sciences (SLU), Lennart Hjelms väg 9, P.O. Box 7014, 75007 Uppsala, Sweden

Correspondence to: Marie Spohn (marie.spohn@slu.se)

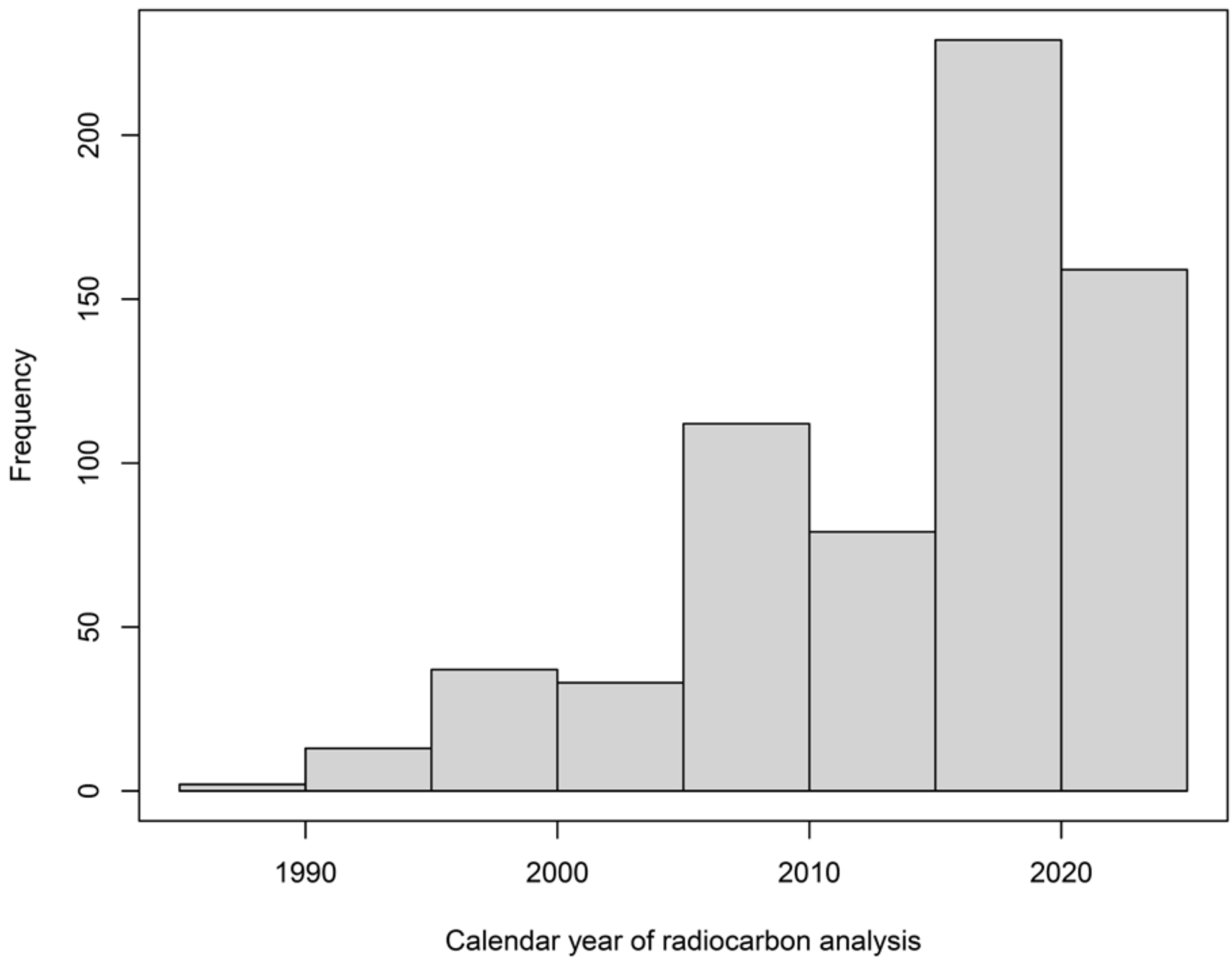

Figure S1 Frequency of the calendar years of radiocarbon analysis (N = 1430).

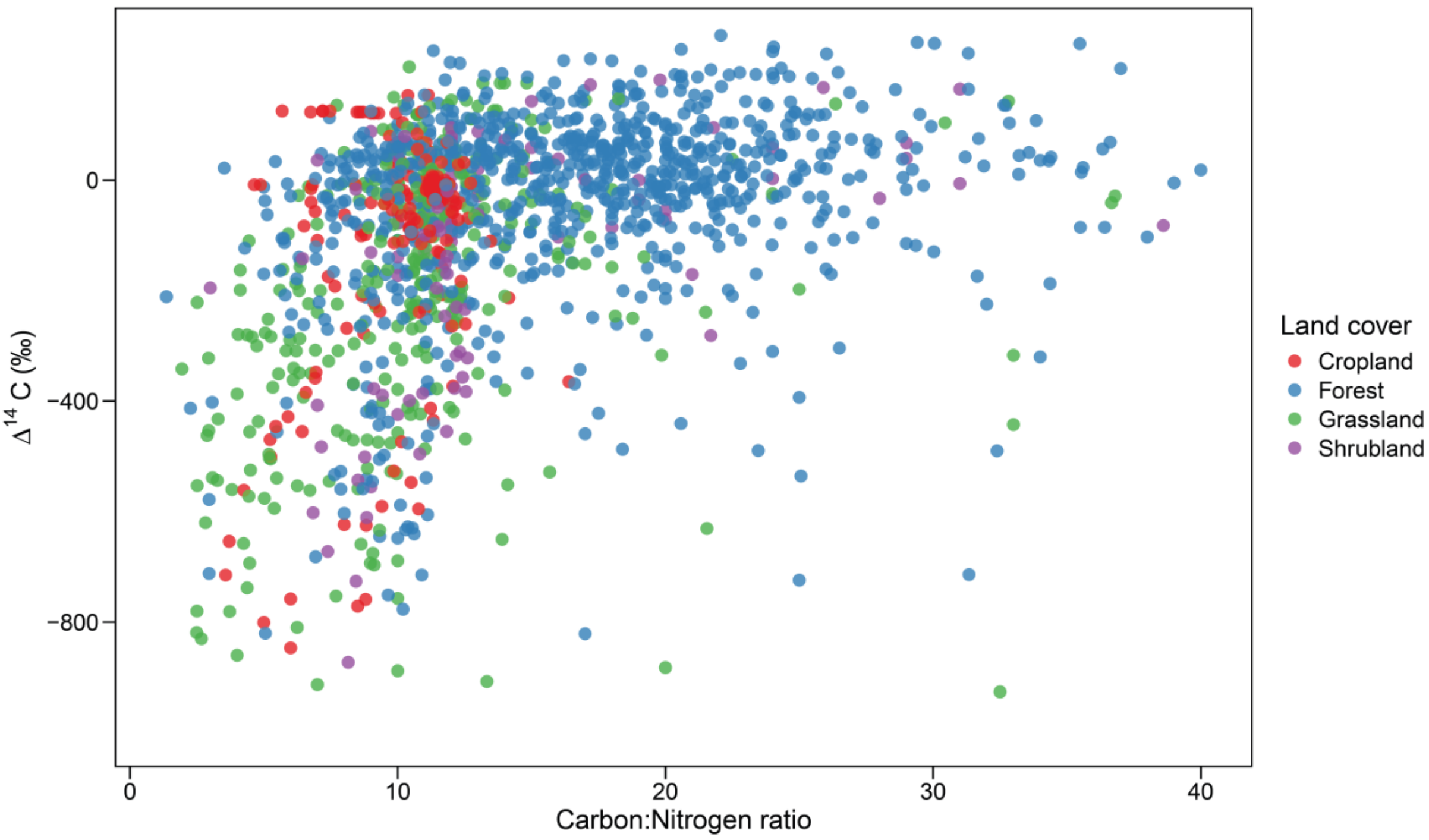

Figure S2 $\Delta^{14}C$ as a function of the C:N ratio. Colors indicate land cover.

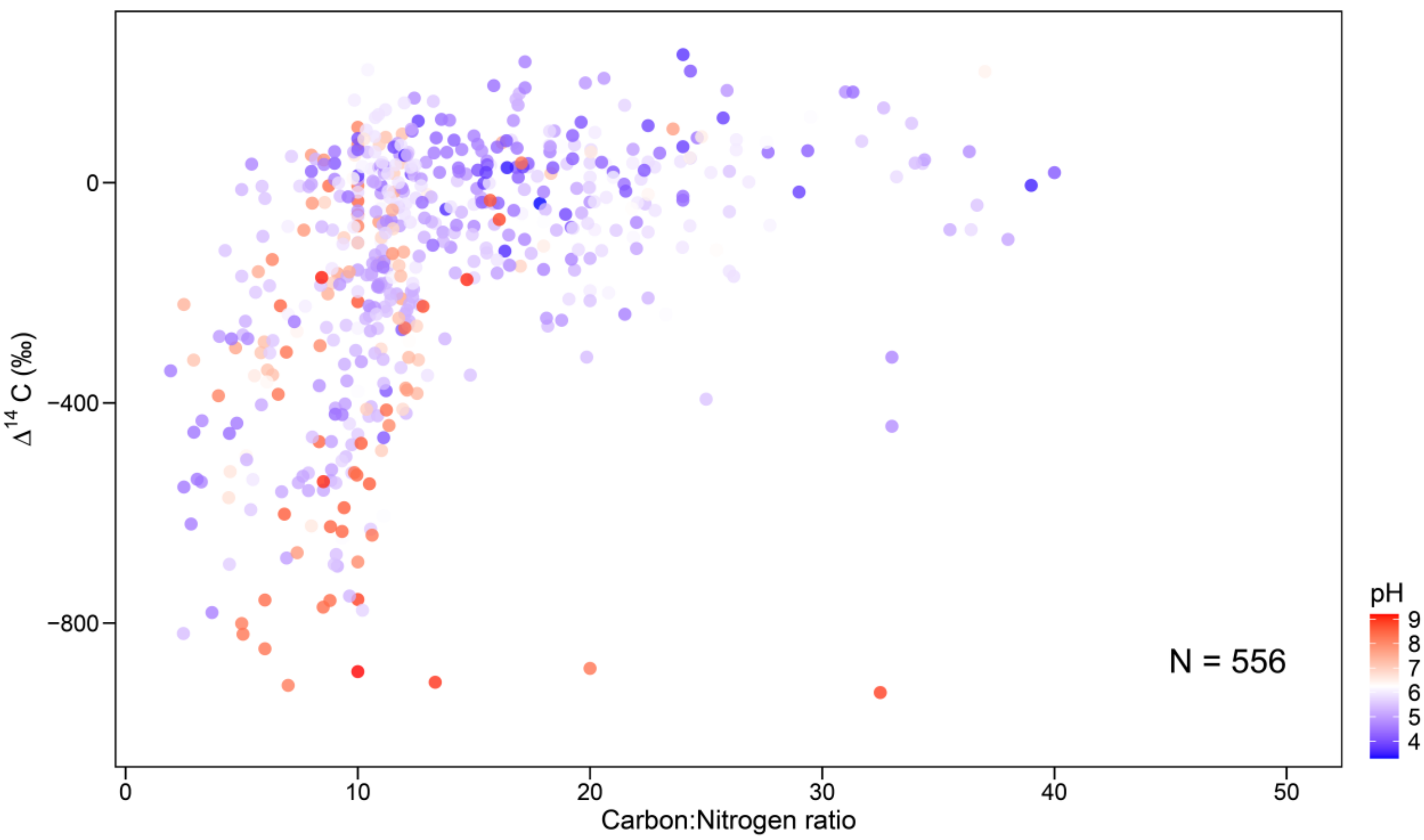

Figure S3 Δ^{14}C as a function of the C:N ratio. Colors indicate soil pH (determined in water). N indicates the number of observations.

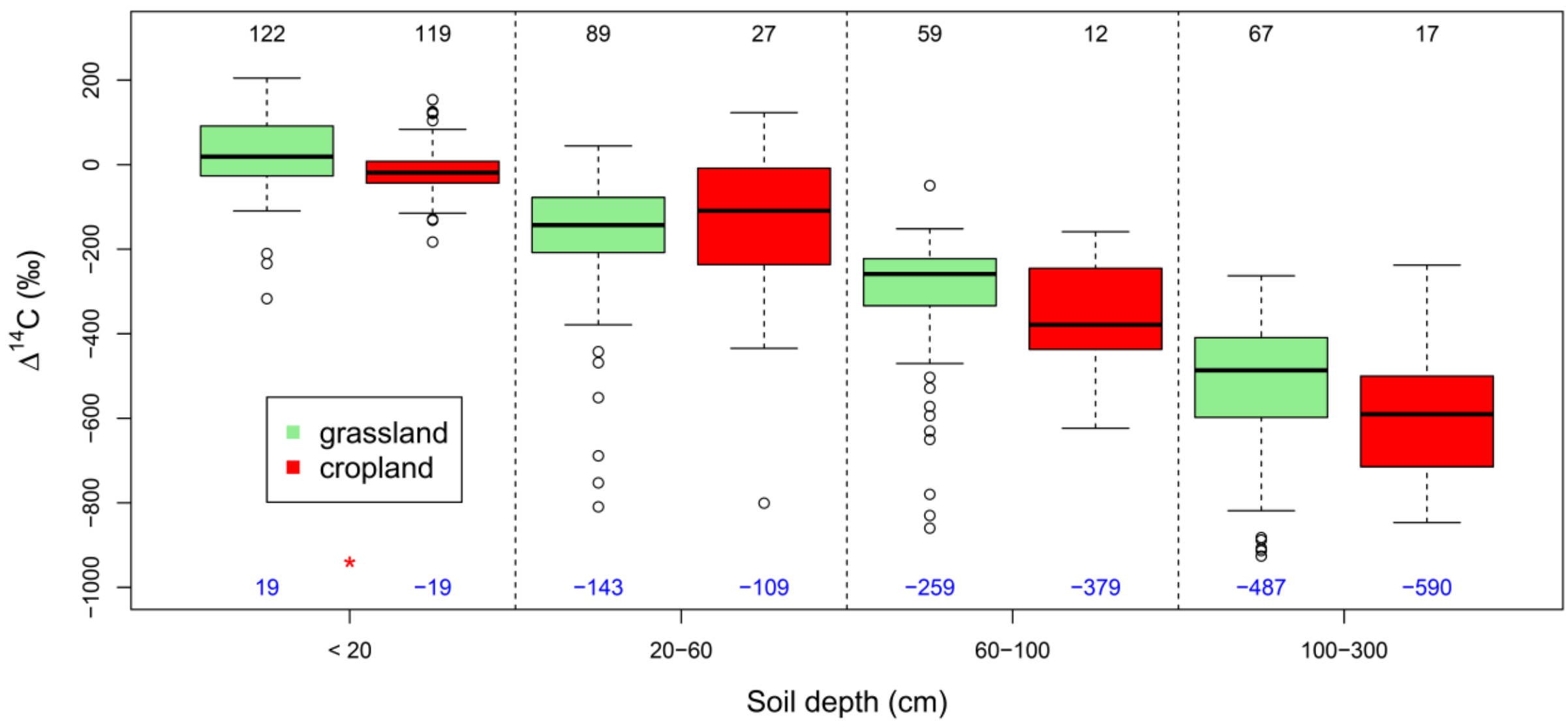

Figure S4 The $\Delta^{14}C$ in different soil depth increments in grasslands and croplands. The black numbers indicate the numbers of observation. The blue numbers depict medians. The 25-% and 75-% percentile define the box and the 10-% and 90-% percentiles define the bars in the boxplots. The red asterisk indicates a significant difference between grasslands and croplands.
